# Multiple imputation and pooling strategies for handling wide-format missing data in latent growth curve modeling

**DOI:** 10.3389/fpsyg.2026.1614844

**Published:** 2026-03-18

**Authors:** Fan Jia, Yueqi Yan

**Affiliations:** Psychological Sciences, University of California, Merced, Merced, CA, United States

**Keywords:** latent growth curve modeling, long format, missing data, multilevel imputation, pooling, wide format

## Abstract

Latent growth curve modeling (LGCM), commonly employed in psychological sciences, often encounters the challenge of missing data, which introduce difficulties into the modeling process. While the full information maximum likelihood (FIML) is the dominant missing data handling technique in practice, an alternative class of techniques, multiple imputation (MI), may be preferable in certain scenarios. With the recent increasing attention shine lights on multilevel MI, we conducted a simulation study examining both standard wide-format (MI-wEMB and MI-wFCS) and multilevel long-format MI methods (MI-llMLM and MI-lqMLM) in comparison with FIML under various missing data conditions and model specifications. Our results indicate that while FIML generally produced unbiased estimates and accurate confidence intervals (CIs), most MI methods demonstrated comparable performance across most conditions. However, the proportion of missing data played a notable role, affecting the performance of the methods to varying extents. We also examined the variation in pooling strategies for the likelihood ratio test (LRT) statistic. The results showed that pooling strategies did not exhibit significant differences in performance, but long-format MI methods displayed concerningly conservative Type I error rates (near zero), regardless of the pooling strategy used. The study further revealed that when the analysis model was misspecified, FIML still maintained the highest power, followed by MI-wEMB, MI-wFCS, and MI-lqMLM. Sample size and missing data proportion had the most significant impact on power. Our findings provide practical guidance for researchers in selecting appropriate missing data approaches for LGCM.

## 1 Introduction

Latent growth curve modeling (LGCM) is widely used in psychological sciences to understand changes over time. It uses latent constructs to estimate the average coefficients of trajectories, e.g., latent intercept and slopes, accounting for individual variations in these coefficients. Although the multilevel modeling (MLM) approach also investigates the growth trajectory of repeated-measures outcomes of interests, LGCM has some advantages over it. For example, LGCM is a structural equation modeling-based approach, and therefore, it explicitly accounts for measurement errors to test the theoretical models. In addition, LGCM enables researchers to investigate relationship among latent growth components (i.e., latent intercept and slopes) and other variables (e.g., covariates and distal outcomes).

Since missing data has been an inevitable issue in longitudinal studies, researchers who employ LGCM must select appropriate methodological approaches to address this challenge. Typical issues caused by missing data are biased parameter estimates, distorted model fit, and power loss (Enders, 2022). Traditional missing data handling approaches, such as listwise deletion and single imputations, have been considered ineffective and problematic. The state-of-arts missing data techniques, such as full information maximum likelihood (FIML) and multiple imputation (MI), have gained increasing popularity in recent decades. FIML is wildly recognized in the fields of latent variable analysis, including LGCM. Previous research revealed when the multivariate normality assumption holds, FIML could produce unbiased parameter estimates and appropriate test statistics, taking into account missing data simultaneously (Collins et al., 2001; Enders and Bandalos, 2001; Rubin, 1987). In contrast, MI is a family of approaches that addresses missing data and perform data analysis in separate steps. While FIML is often the default approach for handling missing data in the LGCM, MI offers several important advantages in many applied research settings. For example, MI can readily accommodate a large number of auxiliary variables that are not part of the analysis model but improve missing data recovery, whereas incorporating numerous auxiliary variables with FIML can be cumbersome and computationally challenging (Graham, 2003; Savalei and Bentler, 2009). In addition, with complex models or complicated data structures, FIML may fail to converge or become computationally infeasible (Savalei and Bentler, 2009). In contrary, MI can often overcome these limitations because the estimation is conducted on completed data sets following imputation (Enders and Mansolf, 2018; Gottschall et al., 2012). MI also offers greater flexibility in specifying imputation models, allowing it to better accommodate nonnormal or nonlinear relationships (Asparouhov and Muthén, 2010; White et al., 2011; Jia and Wu, 2022). This article therefore focuses on conditions under which both FIML and MI are practically applicable and directly comparable, in order to examine their relative performance in a controlled setting. Although MI’s primary advantages often arise in scenarios where FIML is difficult or impossible to apply, establishing their comparative behavior under shared feasible conditions provides an important foundation for understanding their respective strengths and limitations.

Despite its advantages, MI is less straightforward than FIML. It generally requires three steps: imputation (i.e., generating multiple complete data sets with missing values filled in), analysis (i.e., fitting multiple imputed data sets into the target analysis model), and pooling (i.e., combining the analysis results from these multiple imputed data sets). To ensure unbiased parameter estimates, each step requires to implement appropriate approaches. Generally speaking, the imputation model should align with the research context, the nature of the missing data, and the underlying assumptions of missing data mechanism (we will describe it in the next section). For longitudinal data, the process for selecting an appropriate imputation strategy can be even more intricate. Specific to longitudinal studies, data can be organized in either a wide format or long format. In the wide format, observations across measurement occasions are presented in separate variables. In such data structure, each row stores all observational data for one individual but with a greater number of columns. In the LGCM framework, the most common practice is to perform both imputation and a subsequent data analysis in the wide format.

The long format involves vertically stacking the observations from multiple measurement occasions for each individual, with an additional variable indicating the measurement occasion. This data format is commonly used within the MLM framework. For data organized in long format, several imputation methods suitable for the multilevel data structure have been proposed, especially for cross-sectional analysis (e.g., students clustered within classroom; Audigier and Resche-Rigon, 2018; Carpenter et al., 2011; Enders et al., 2020; Quartagno and Carpenter, 2016, 2019; Resche-Rigon and White, 2018; van Buuren and Groothuis-Oudshoorn, 2011; Zhao and Schafer, 2015). Conceptually, the multilevel imputation approach is appropriate for the longitudinal data for a LGCM model, i.e., repeated measures inherently nested within individuals. However, limited research has examined their performance with such longitudinal data structures. One complication is that such investigation requires data transformation between wide and long formats. Specifically, one needs to convert original data to long format for the multilevel imputation process, then revert imputed data back to wide format to accommodate the requirements of the latent growth analysis model. Given the growing awareness in multilevel MI methods for longitudinal designs and recent software advancements in the MLM framework (e.g., Enders et al., 2020), it is timely and relevant to evaluate how these conceptually appropriate multilevel MI methods (a.k.a., *long-format MI*) perform when applied to repeated-measures data in LGCM, compared to standard, single-level *wide-format MI*.

Another recent methodological advancement in MI further motivated this study. While parameter estimates and standard errors can be directly pooled using Rubin’s (1987) rules, strategies for pooling likelihood ratio test (LRT) statistics and practical fit indices have received comparatively little attention. Enders (2022) summarizes two strategies for pooling LRT: D_2_ (Li et al., 1991) and D_3_ (Meng and Rubin, 1992), which have been evaluated in a few simulation studies (e.g., Enders and Mansolf, 2018; Liu and Sriutaisuk, 2020) under different scenarios. In more recent work, Chan and Meng (2022) proposed a new LRT statistic pooling strategy, which has been denoted as D_4_ and proven at least as effective as D_3_ while offering greater efficiency (Grund et al., 2023). To our knowledge, none of these pooling approaches has been evaluated within the LGCM framework.

This current article aims to provide researchers with guidance on the best practice in handling missing data using MI when performing a LGCM, including recommendations for selecting appropriate imputation methods (e.g., wide-format vs. long-format) and optimal pooling strategies for model fit evaluation. The remaining of the article is organized as follows. We first introduce the basics and principals of LGCM, and then discuss missing data mechanisms and highlight the difference among commonly used missing data techniques, with a focus on MI. We then delve into the imputation phase in MI, introducing imputation algorithms and imputation models that are suitable for scenarios in the LGCM framework. Additionally, the pooling phase is reviewed, with an emphasis on the pooling strategies for the LRT statistic. Moving forward, we present a simulation study that compared the combinations of these imputation and pooling strategies with FIML. Finally, we provided an empirical example and conclude with practical guidance on implementing these strategies in MI in the context of LGCM.

## 2 Latent growth curve modeling

A classic application of LGCM is to analyze continuous repeated-measures data collected from a group of individuals and estimate intraindividual (within-person) growth over time [i.e., latent intercept and latent slope(s)], while accounting for variability at interindividual (between-person) levels (Kohli and Harring, 2013; Bollen and Curran, 2006). A latent intercept captures the initial level; and latent slope(s) usually represent a growth pattern as a polynomial function of time, such as linear, quadratic, cubic, etc. These two types of latent growth factors are measured by their mean levels, individual variances, and covariances between them (Depaoli et al., 2023). The repeated measures are indicators of latent growth factors, linked by factor loadings. A typical latent quadratic model, for example, can be presented in matrix form in Equation 1.


$$y=\Lambda_{y}\times \eta +\varepsilon ,$$

$$[\begin{array}{c} Y_{1}\\ Y_{2}\\ Y_{3}\\ \vdots \\ Y_{T} \end{array}]=[\begin{array}{ccc} 1 & t_{1} & t_{1}^{2}\\ 1 & t_{2} & t_{2}^{2}\\ 1 & t_{3} & t_{3}^{2}\\ \vdots & \vdots & \vdots \\ 1 & t_{T} & t_{T}^{2} \end{array}]\times [\begin{array}{c} \eta_{I}\\ \eta_{S1}\\ \eta_{S2} \end{array}]+[\begin{array}{c} \varepsilon_{1}\\ \varepsilon_{2}\\ \varepsilon_{3}\\ \vdots \\ \varepsilon_{T} \end{array}],$$ (1)

where $t_{j}$ is time code for the *j*^th^ measurement occasion; $Y_{j}$ represents a vector of outcome for all individuals at the *j*^th^ measurement occasion; and $\varepsilon_{j}$ is a residual vector at the *j*^th^ measurement occasion (*j* = 1, 2, … *T*). The latent intercept, latent linear slope, and latent quadratic slope are denoted by $\eta_{I}$ , $\eta_{S1}$ , and $\eta_{S2}$ , respectively. For a linear model, the model is a reduced form of Equation 1, with the $\Lambda_{y}$ matrix only containing the first two columns, and η matrix only containing $\eta_{I}$ and $\eta_{S1}$ .

The model fit evaluation is commonly based on the likelihood ratio test (LRT) statistics, which assesses the difference between the model implied and observed covariance matrices and mean vectors. The LRT statistic, denoted *T*, follows a $\chi^{2}$ distribution assuming data are normally distributed and can be written as follows.


$$T=-2[l(\hat{\theta })-l(\tilde{\beta })],$$ (2)

where $l(\hat{\theta })$ is the maximized log-likelihood under the hypothesized model, and $l(\tilde{\beta })$ is the corresponding log-likelihood of the saturated model (Bollen, 1989).

## 3 Multiple imputation in missing data handling

Data can be missing during the data collection process through various ways. Rubin (1976) summarized three primary missing data mechanisms, including missing completely at random (MCAR), missing at random (MAR), and missing not at random (MNAR). MAR occurs when probability of data being missing on a variable is only related to the observed variables. MCAR is sometimes considered as a special case of MAR; the former refers to the situation where the probability of data being missing on a variable is unrelated to its values or any other observed variables in the dataset. These two mechanisms are commonly referred to as “ignorable missingness” when the condition of “distinct parameters” holds. That means, the parameters governing the distribution of the data are distinct from those governing the missing data process. In an LGCM, this implies that the joint likelihood can be separated into two parts: a part for the LGCM and a part for the missingness mechanism. Because the missingness part does not contain information about the growth parameters, it does not need to be modeled in order to obtain valid estimates of the growth parameters. In contrast, MNAR, represents a “non-ignorable” missingness scenario where probability of data being missing on a variable is determined by unobserved values themselves, demanding more intricate methodological approaches for an effective solution. A common type of missingness in longitudinal studies, called *attrition*, happens when participants drop out from a study after initially contributing data in the early measurement occasions. Attrition at later occasions is likely to be informed by the data available from the preceding occasions (Allison, 2001), in which case, the missing data due to attrition can reasonably be assumed to be MAR.

In situations where an “ignorable” missing data mechanism is present (i.e., MCAR or MAR), modern missing data techniques, such as full information maximum likelihood (FIML) or multiple imputation (MI), can effectively recover the missing information. FIML is an approach that estimates the parameters of a statistical model and handle missing data simultaneously. It is based on an iterative process that aims to find the values maximizing the overall log-likelihood, while accounting for the patterns of missing data (Enders, 2022). MI, in contrast, typically handle missing data through three steps. In MI, missing values are first imputed multiple times based on a certain parametric or nonparametric model, creating multiple complete datasets (the imputation step). These imputed datasets are subsequently used for the target statistical analysis individually (the analysis step). Then, the results of all the multiple imputed datasets are pooled to generate the final result (the pooling step; Rubin, 1987). This multi-step approach could avoid potential convergence problems FIML may have with complex data or models and enables flexibility in incorporating auxiliary variable and handling various types of missing data. However, the implementation of MI could be more challenging than FIML as it requires more complex methodological considerations. For example, researchers need to make decisions on the choices of (1) imputation algorithm, (2) imputation model, and (3) pooling strategies for different types of statistics, especially for the LRT statistic and model fit indices.

### 3.1 Imputation algorithms

Imputation algorithms refer to the procedures used to fill in missing data in a dataset. The most well-known imputation algorithms in the missing data literature are distinguished by whether missing values are imputed based on a multivariate distribution or a series of conditional univariate distributions. The first type of algorithms includes the joint modeling (JM; Schafer, 1997) and expectation–maximization with bootstrapping (EMB; Honaker et al., 2011). Both algorithms assume data follow a multivariate normal distribution and are theoretically equivalent (Enders, 2011). The JM algorithm uses an iterative Markov Chain Monte Carlo (MCMC) procedure that alternates between drawing imputations for missing values and drawing parameters from their conditional posterior distributions. The EMB algorithm creates a specified number of bootstrapped samples (e.g., 5,000) and then applies the expectation–maximization algorithm to obtain maximum likelihood estimates of the mean vector and covariance matrix for each bootstrapped sample. To create each imputed dataset, the algorithm randomly selects one set of parameter estimates from the bootstrapped samples and uses these estimates to impute the missing values.

Another group of imputation algorithms are commonly known as Fully Conditional Specification (FCS; a.k.a., MI by chained equations or MICE; van Buuren et al., 2006; van Buuren and Groothuis-Oudshoorn, 2011), in which imputation is conducted on a variable-by-variable basis, conditional on all other variables in the dataset. This process is repeated iteratively until missing values for all variables are filled in. The entire procedure is then repeated multiple times to generate *m* imputed datasets. The MICE process can adapt flexibly to accommodate specific characteristics of different incomplete variables.

### 3.2 Imputation models

Imputation models refer to the statistical models used to estimate the missing values in a dataset. For example, EMB fills missing data through random draws from a multivariate normal distribution using estimated parameters, while FCS imputes missing values using a series of univariate conditional models, each based on the distribution appropriate for the variable being imputed, such as linear regression with normal residuals assumed. In the context of LGCM, data should be organized in the wide format, where the repeated measure at *j*^th^ measurement occasion is stored as an individual variable ( $Y_{j}$ ) in the dataset. If one chooses to maintain this wide data structure during imputation, either standard EMB or FCS can be performed, depending on the chosen distribution model, i.e., multivariate normal distribution for EMB or models under univariate normal distributions for FCS. Imputation using EMB can be implemented through R package Amelia (Honaker et al., 2011). R package mice (van Buuren and Groothuis-Oudshoorn, 2011) or the software Blimp (Keller and Enders, 2023) can be used for FCS imputation. In this article, we refer to the imputation strategy that preserves the wide format of data as the wide-format imputation.

In contrast, to account for the dependency of repeated measures within individuals in longitudinal studies, various multilevel imputation approaches, built on different multilevel imputation models, have developed over time and recently gained significant attention in methodological research (e.g., Audigier and Resche-Rigon, 2018; Carpenter et al., 2011; Enders et al., 2020; Quartagno and Carpenter, 2016, 2019; Resche-Rigon and White, 2018; Zhao and Schafer, 2015). We refer to these types of imputation strategies as the *long-format* imputation, as they impute data while stacking the lower-level records for each upper-level unit, and account for the multilevel nature of data through a restricted model. Grund et al. (2018) systematically evaluated several long-format MI approaches that account for clustering data structure (but not necessarily longitudinal data) within multilevel modeling frameworks. Their evaluation compared multivariate-based and FCS imputation methods while varying analysis model complexity, including the incorporation of random effects and nonlinear terms. Based on their simulation results, long-format multilevel MI was recommended in the majority of scenarios. In contrast, Huque et al. (2018) compared a broader range of wide-format and long-format imputation methods (implemented in a broader range of software packages) in longitudinal linear regression models, including a panel model and a multilevel random-intercept model. They found that the wide-format MI approaches outperformed the long-format approaches the models examined. Ender (2022) demonstrated both wide-format and long-format imputation strategies with a single sample using the Blimp software (Keller and Enders, 2023) in comparison with FIML. It shows that the parameter estimates of a latent growth curve model from wide-format imputation is generally comparable but a bit “nosier” than the other two methods.

The contradictory recommendations from Grund et al. (2018) and Huque et al. (2018) regarding the performance of wide-format versus long-format imputation methods likely arise from differences in their modeling frameworks. A possible explanation of the discrepancies could be that Grund et al. (2018) focused on clustered data scenarios with complex multilevel analysis models that included random slopes, nonlinear effects, and cross-level interactions, where long-format multilevel MI better matched the analysis model’s clustered structure and thus enhanced estimation in variance components and interaction estimates. In contrast, Huque et al. (2018) examined longitudinal designs with simpler analyses, such as linear mixed models with random intercepts only or panel regression, where wide-format methods more flexibly captured unstructured correlations without the risk of overparameterization in multilevel imputation, leading to better or comparable performance in bias and coverage. This context-dependence underscores that neither wide-format nor long-format imputation is universally superior. Instead, the optimal choice of imputation method should be guided primarily by the degree of congeniality (alignment) between the imputation model and the target analysis model, as well as by the specific characteristics of the data and analysis.

Inspired by the Enders’ (2018) non-simulation-based comparison within the LGCM framework and the recent software development (such as Blimp; (Keller and Enders, 2023), the *primary objective* of this article is to systematically investigate the relative performance of wide-format versus long-format imputation methods in the context of LGCM, where the target analysis inherently requires the data to be structured in wide format. Technically, MI in long-format for a LGCM requires data transformation from wide to long formats prior to imputation, and back-transformation for performing a LGCM subsequently. Given that model fit evaluation is a critical component of LGCM, as in other latent variable models, we also assessed the performance of these approaches in estimating the LRT statistic, in addition to their accuracy and precision in estimating model parameters.

### 3.3 Pooling strategies

Regardless of the imputation methods, after the imputation and analysis steps, the results obtained from each of imputed datasets need to be pooled together. It is straight forward to pool the parameter estimates and their standard errors (Rubin, 1987). A final parameter estimate is simply the arithmetic mean of parameter estimates across imputed datasets. The pooled standard error of a parameter estimate ( $V_{\text{pooled}}$ , Equation 3) can be written as a function of within-imputation variance ( $V_{W}$ ) and between-imputation variance ( $V_{B}$ ) with *m* imputed datasets.


$$V_{B}=\frac{1}{m-1}\sum_{i=1}^{m}{\left(\theta_{i}-\bar{\theta }\right)}^{2},$$

$$V_{W}=\frac{1}{m}\sum_{i=1}^{m}V_{i},$$

$$V_{\text{pooled}}=V_{W}+V_{B}+\frac{V_{B}}{m}.$$ (3)

Pooling the LRT statistic statistics across imputed datasets, however, is a more intricate process. Li et al. (1991) proposed an approach that pools *m* Wald test statistics to generate a final significance test statistic, which is referred to as the D_2_ approach in the missing data analysis literature (Enders, 2011). Although developed on a basis of the Wald test statistic, D_2_ can be applied to LRT, which is asymptotically equivalent to a Wald test (Buse, 1982). Let ${\bar{T}}_{W}$ represents the average of *m* Wald test statistics, and the D_2_ statistic can be defined as:


$$D_{2}=\frac{{\bar{T}}_{W}\;k^{-1}-(m+1){\left(m-1\right)}^{-1}\mathrm{ARI}V_{2}}{1+\mathrm{ARI}V_{2}},$$

where *k* is the degree of freedom of LRT (i.e., the difference in the number of free parameters between the hypothesized and the saturated models); and $\mathrm{ARI}V_{2}$ is an estimate of the average relative increase in variance due to missing data. The D_2_ approach can pool LRT statistics from *m* imputed datasets (Jorgensen and Rosseel, 2025) and approximates an *F* distribution across these datasets for significance testing.

The D_3_ statistic, developed by Meng and Rubin (1992), involves three steps. First, it averages the LRT statistics from *m* imputed datasets to obtain ${\bar{T}}_{\mathrm{LR}}$ .


$${\bar{T}}_{\mathrm{LR}}=\frac{1}{m}\;\sum_{i=1}^{m}T_{i},$$ (4)

where the LRT statistics ( $T_{i})$ is the same as in Equation 2 for the *i*^th^ imputed dataset. Second, the analysis model is fit to each imputed dataset with the parameters fixed at the pooled values, and the average LRT statistic ( ${\bar{T}}_{\text{Constrianed}}$ ) is computed in the same way as in Equation 4. The final step is to calculate the pooled likelihood ratio test statistics as follows:


$$D_{3}=\frac{{\bar{T}}_{\text{Constrianed}}}{k(1+\mathrm{ARI}V_{3})},$$

where $\mathrm{ARI}V_{3}$ is also an estimate of the average relative increase in variance due to missing data, which is a function of ${\bar{T}}_{\mathrm{LR}}$ , ${\bar{T}}_{\text{Constrianed}}$ and *m*.

The D_4_ approach represents the most recent advancement in the field of test statistic pooling strategies (Chan and Meng, 2022; Grund et al., 2023). This approach first stacks all the imputed dataset on top of one another and then fit the analysis model to the “stacked” data to obtain the likelihood ratio test statistic ( $T_{\text{stacked}}$ ) as described in Equation 1. The D_4_ statistic can be written as follows:


$$D_{4}=\frac{{\bar{\bar{T}}}_{\text{Stacked}}}{k[1+\max(\mathrm{ARI}V_{4},0)]},$$

where ${\bar{T}}_{\text{Stacked}}$ is computed as $T_{\text{stacked}}$ divided by *m*; and $\mathrm{ARI}V_{4}$ is another estimate of the average relative increase in variance.

The methodological research on these pooling strategies remains limited. Liu and Sriutaisuk (Liu and Sriutaisuk, 2020) only focused on performance of D_2_ with ordinal items in the confirmatory factor analysis. Enders and Mansolf (2018) revealed that D_3_ was comparable to FIML in estimating LRT statistic in a structural equation model. In the meanwhile, D_3_ was criticized for its failure to remain invariant under equivalent parameterizations of the same model and for its inability to ensure a non-negative value (Chan and Meng, 2022; Enders and Mansolf, 2018). Chan and Meng (2022) claimed that D_4_ could perform at lease as well as D_3_, as it is invariant to re-parameterization and is assured to have a non-negative value. It was also expected to be more computationally efficient as it does not involve re-evaluating the likelihood function as D_3_ does. Less research has focused this most recently proposed pooling strategy, D_4_. So far, none of these pooling strategies, a critical component of MI for latent variable models, have been evaluated in the LGCM framework. Therefore, the *secondary objective* of this article is to evaluate the performance of these pooling strategies when preforming various MI approaches in the LGCM.

## 4 Simulation

### 4.1 Design

**Figure 1:**
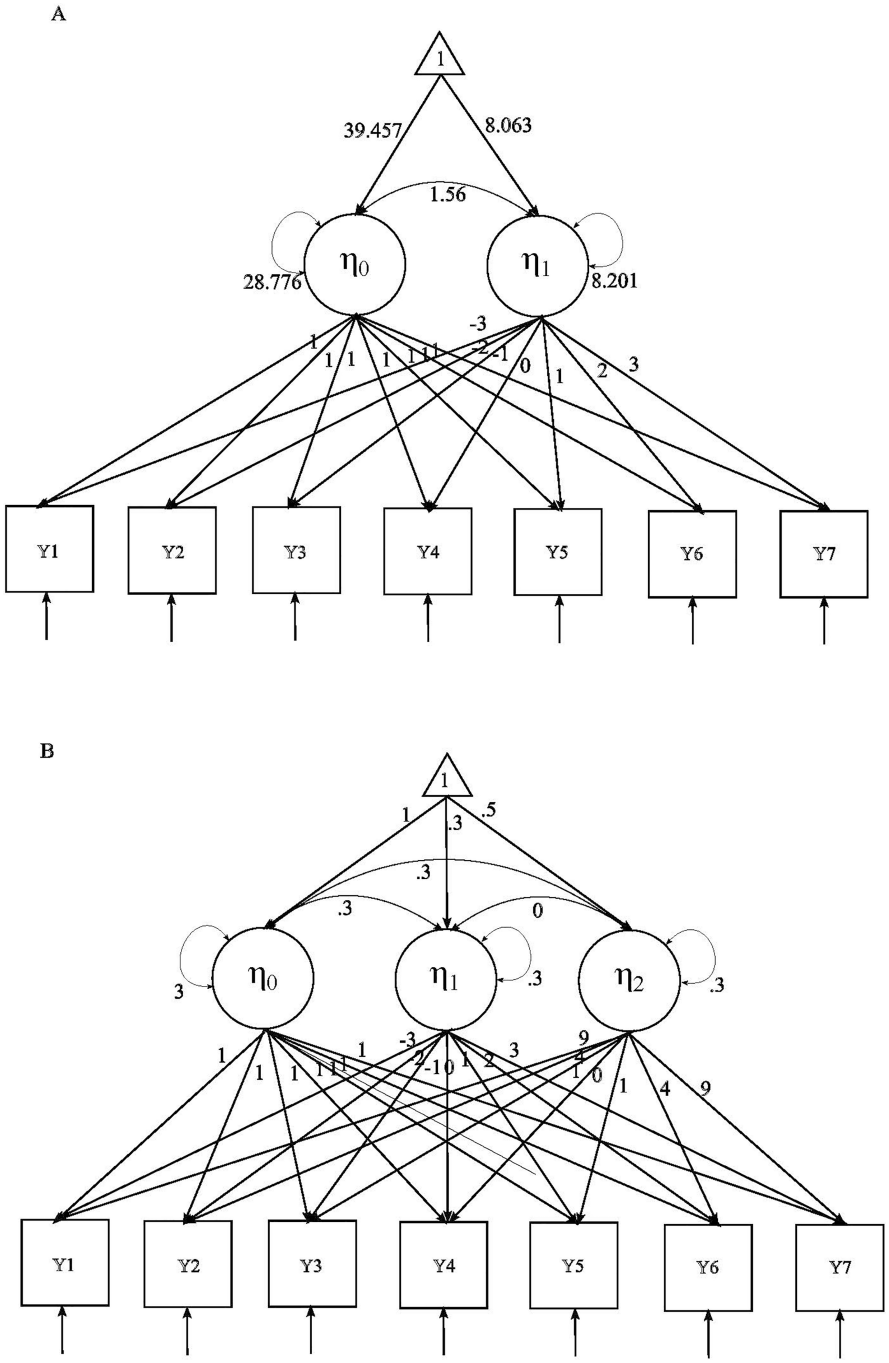
Population models: **(A)** linear latent curve growth model; **(B)** quadratic latent curve growth model.

We conducted three simulation studies to evaluate the performance of various imputation and pooling strategies within the LGCM framework, with FIML serving as the reference method. Although MI’s practical advantages commonly arise in contexts where FIML is not feasible (e.g., large number of auxiliary variables, complex models, or non-normal data), we deliberately examined conditions where both methods are practically feasible and directly comparable. Because understanding MI’s performance in these scenarios could provide critical insight into its potential benefits in more complex situations where FIML may be impractical or unavailable. In Study 1, data were generated using a linear latent curve growth model with 7 measurement occasions (see Figure 1A), with population parameters slightly adjusted from those in Biesanz et al. (2004). The analysis model used was the same as the generation model. Five missing data techniques were evaluated in this study:

1. FIML;
2. MI-wEMB (MI with *w*ide-format data through EMB);
3. MI-wFCS (MI with *w*ide-format data through FCS);
4. MI-llMLM (MI with *l*ong-format data and *l*inear *M*ulti*L*evel imputation *M*odel);
5. MI-lqMLM (MI with *l*ong-format data and *q*uadratic *M*ulti*L*evel imputation *M*odel).

MI-wEMB refers to the wide-format EMB imputation implemented using the R package Amelia (Honaker et al., 2011), which assumes a multivariate normal distribution and imputes missing data through the EMB algorithm. MI-wFCS is also a wide-format imputation method; however, it employs a fully conditional specification (FCS) algorithm that imputes missing values on a variable-by-variable basis. MI-wFCS in the present simulation was conducted using Blimp (Keller and Enders, 2023), which employs the Bayesian FCS algorithm (10,000 iterations with 5,000 burn-ins), and imputes missing data through a round-robin sequence of regression models. Both MI-llMLM and MI-lqMLM are long-format imputation methods implemented in Blimp (Keller and Enders, 2023) that employ fully Bayesian JM algorithm (10,000 iterations with 5,000 burn-ins), and explicitly account for the multilevel structure of the data. MI-llMLM was selected to align with the linear multilevel analysis model. MI-lqMLM was included to represent a scenario in which researchers apply a more complex imputation model (e.g., quadratic) before fitting a simpler nested analysis model (e.g., linear) when there is uncertainty about the functional form of the trajectory, as recommended by Enders (2018). In Blimp, missing values were imputed using a model-based approach, whereby a multilevel growth curve model (linear or quadratic, with random slopes) was specified as the imputation model. For all imputation methods, 100 imputed datasets were generated.

In Study 2 we used a quadratic latent curve growth model with 7 measurement occasions (see Figure 1B) for data generation, with adapted population values from Wu and West (2010). The analysis model was the same as the generation model. Four missing data techniques were examined: FIML, MI-wEMB, MI-wFCS, and MI-lqMLM.

Study 3 mirrored the Study 2 in terms of data generation and missing data handling approaches. However, this study employed a slightly misspecified analysis model, in which the covariance between the intercept and quadratic slope was incorrectly fixed at zero. For all MI methods in the three studies, the parameter estimates, and standard errors were pooled following the Rubin’s (1987) Rules; while the test statistics were pooled using the three aforementioned strategies: D_2_, D_3_, and D_4_. The inclusion of model misspecification was theoretically and practically motivated. From a theoretical perspective, missing data handling represents only one component of the overall LGCM analysis process, and its performance should be evaluated not only under ideal conditions but also when the analysis model deviates from the true population model. Methodological research in latent variable modeling commonly investigates inferential behavior under model misspecification in order to assess the robustness of estimation and hypothesis testing procedures. From a practical perspective, researchers rarely know the true model and must draw substantive conclusions under some degree of misspecification. Accordingly, Study 3 was designed to evaluate how different multiple imputation and pooling methods influence the inferential performance of the LRT statistic, specifically its statistical power, under various conditions.

Table 1 reported the parameter values in the three population models. For all studies, we chose *N* = 150, 300, and 600 to represent small, medium, and large sample sizes, respectively. Missing data were introduced based on MCAR and MAR with two levels of missing data proportions. The expected missing data proportion for each measurement occasion (*j*) was determined following either of the two linear functions:


$$mp_{\text{samll},j}=.075(j-1),$$

$$mp_{\text{large},j}=.175(j-1),$$

where *j* = 1, 2, …, 7. Therefore, the proportions on the seven time points were (0, 0.05, 0.1, 0.15, 0.2, 0.25, 0.3) for small missing proportions, and (0, 0.1, 0.2, 0.3, 0.4, 0.5, 0.6, 0.7) for large missing proportions. The probability of each value (i.e., the *i*^th^ individual at *j*^th^ occasion) being missing is determined using logistic regression. Specifically,


$$p_{i,j}=\frac{e^{b_{0}+b_{1}Y_{i,\;j-1}}}{1+e^{b_{0}+b_{1}Y_{i,j}-1}},$$ (5)

where *j* = 2, …, 7; and b_0_ varied to achieve the target expected missing data proportion. For MCAR, *b*_1_ was set at 0, indicating the missing data are not determined by any variable. For MAR, *b*_1_ was set at 0.4, defining the effect of $Y_{i,j-1}$ on log-odds ratio of missingness on $Y_{i,j}$ (Enders and Mansolf, 2018).

**Table 1:** Parameter values in population models.

Parameter	Study 1	Study 2	Study 3
Mean of intercept	39.457	1	1
Mean of linear slope	8.063	0.3	0.3
Mean of quadratic slope		0.5	0.5
Variance of intercept	28.776	3	3
Variance of linear slope	8.201	0.3	0.3
Variance of quadratic slope		0.3	0.3
Covariance of intercept and linear slope	1.56	0.3	0.3
Covariance of intercept and quadratic slope		0.3	0.3
Covariance of linear slope and quadratic slope		0	0

In each study, we fully-crossed these factor levels, and for each condition, the performance of each method was evaluated by aggregating results across 500 replications. Data generation and analysis, including FIML, was conducted using the lavaan package (Rosseel, 2012) in R (R core team, 2021). R package lavaan.mi (Jorgensen and Rosseel, 2025) was used for pooling the results.

### 4.2 Outcome measures

#### 4.2.1 Bias

We assessed standardized bias for each parameter estimate in the correct model only. This measure is calculated by taking the difference between the estimated value and the population value and dividing it by the standard deviation of the estimated values across replications. In accordance with Collins et al. (2001), we deemed a standardized bias acceptable when it falls below 0.4.

#### 4.2.2 MSE

Mean squared error (MSE) measures the average squared difference between the estimated value and the population value, providing a quantitative assessment of both the accuracy and precision of a missing data method.

#### 4.2.3 CIC

The 95% confidence interval coverage (CIC) is a proportion of replications, in which the 95% confidence intervals of a certain parameter contain the true population value. CIC is also an valuable tool for evaluating both the accuracy and precision of a quantitative method. A 95% CIC value are usually expected to be higher than 0.90. Following Bradley’s (1978) “liberal criterion,” we considered a CIC “acceptable” if it fell between 0.925 and 0.975.

#### 4.2.4 Type I error rate and power

We finally computed the proportion of replications with a significant LRT statistic (*p* < 0.05) as the Type I error rate in Studies 1 and 2. Because in Studies 1 and 2, the analysis models are correctly specified to match the population model, and therefore the proportion of significant LRT statistic is the estimate of Type I error rate. We considered the Type I error rate to be “accurate” if it was between 0.025 and 0.075 (Bradley, 1978). In contrast, Study 3 introduced model misspecification by constraining the covariance between the intercept and quadratic slope to zero despite a nonzero population value; under this condition, the proportion of significate LRT statistics reflects the statistical power to detect this misspecification.

## 5 Results

### 5.1 Study 1

#### 5.1.1 Parameter estimates

**Figure 2:**
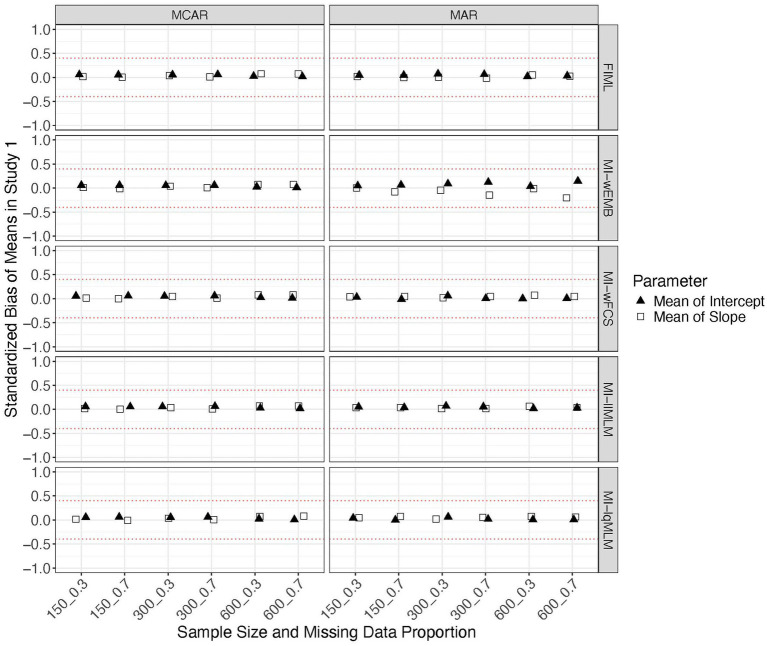
Study 1 results: standardized biases from missing data approaches for the latent means in the linear model.

**Figure 3:**
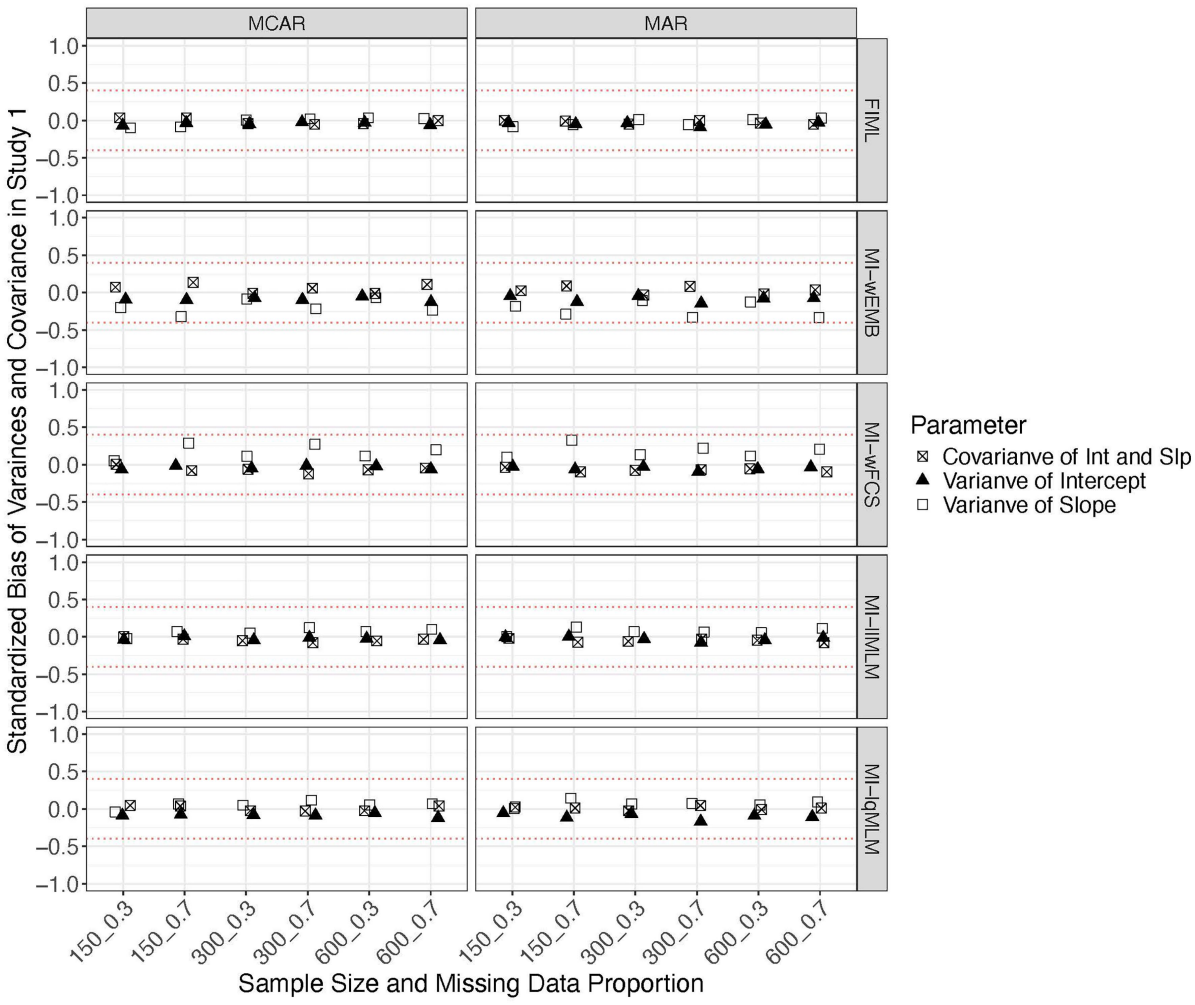
Study 1 results: standardized biases from missing data approaches for the latent variances and covariance in the linear model.

In each condition, we computed standardized biases, MSEs and CICs of parameters for the latent intercept and slope, including means, variances and covariance. Figures 2, 3 show that all methods produced unbiased parameter estimates, especially for the means of the latent factors, regardless of sample size, missing data proportion and mechanism. Comparing among the methods, FIML and MI-llMLM produced most accurate parameter estimates for the variances and covariance.

**Figure 4:**
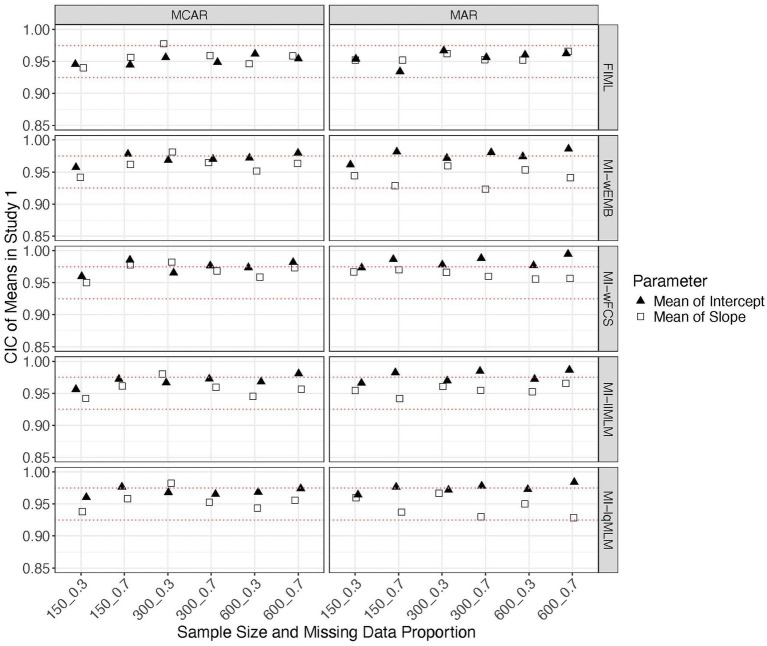
Study 1 results: CICs from missing data approaches for the latent means in the linear model.

**Figure 5:**
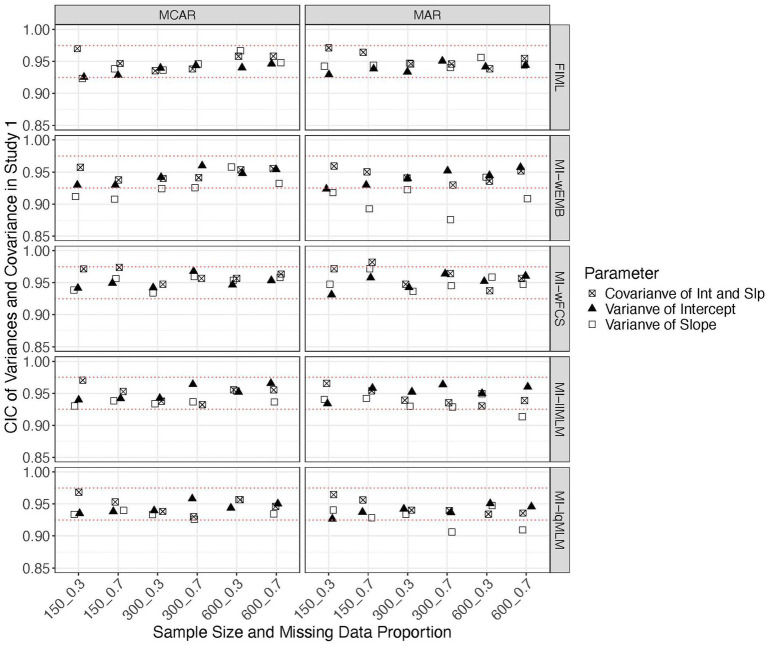
Study 1 results: CICs from missing data approaches for the latent variances and covariance in the linear model.

We found that all methods produced comparable MSEs.^1^ Sample size had a greater impact on the mean and variance of the latent intercept than other parameters. Other factors did not show a noticeable impact on MSE. All CICs are generally within the acceptable range (i.e., >0.90). We found that FIML could stably produce accurate confidence intervals across all conditions for all parameter types (Figures 4, 5). All MI methods produced generally accurate (i.e., between 0.925 and 0.975) CICs for the mean of latent slope (Figure 4), variance of latent intercept and covariance between intercept and slope (Figure 5). The CICs of the mean of intercept can be slightly larger than 0.975, for all MI methods under MAR with the larger missing data proportion (Figure 4). The CICs for variance of slope could be lower than 0.925 for MI-wEMB and MI-lqMLM, under MAR with the larger missing data proportion (Figure 5).

#### 5.1.2 Type I error rate

**Figure 6:**
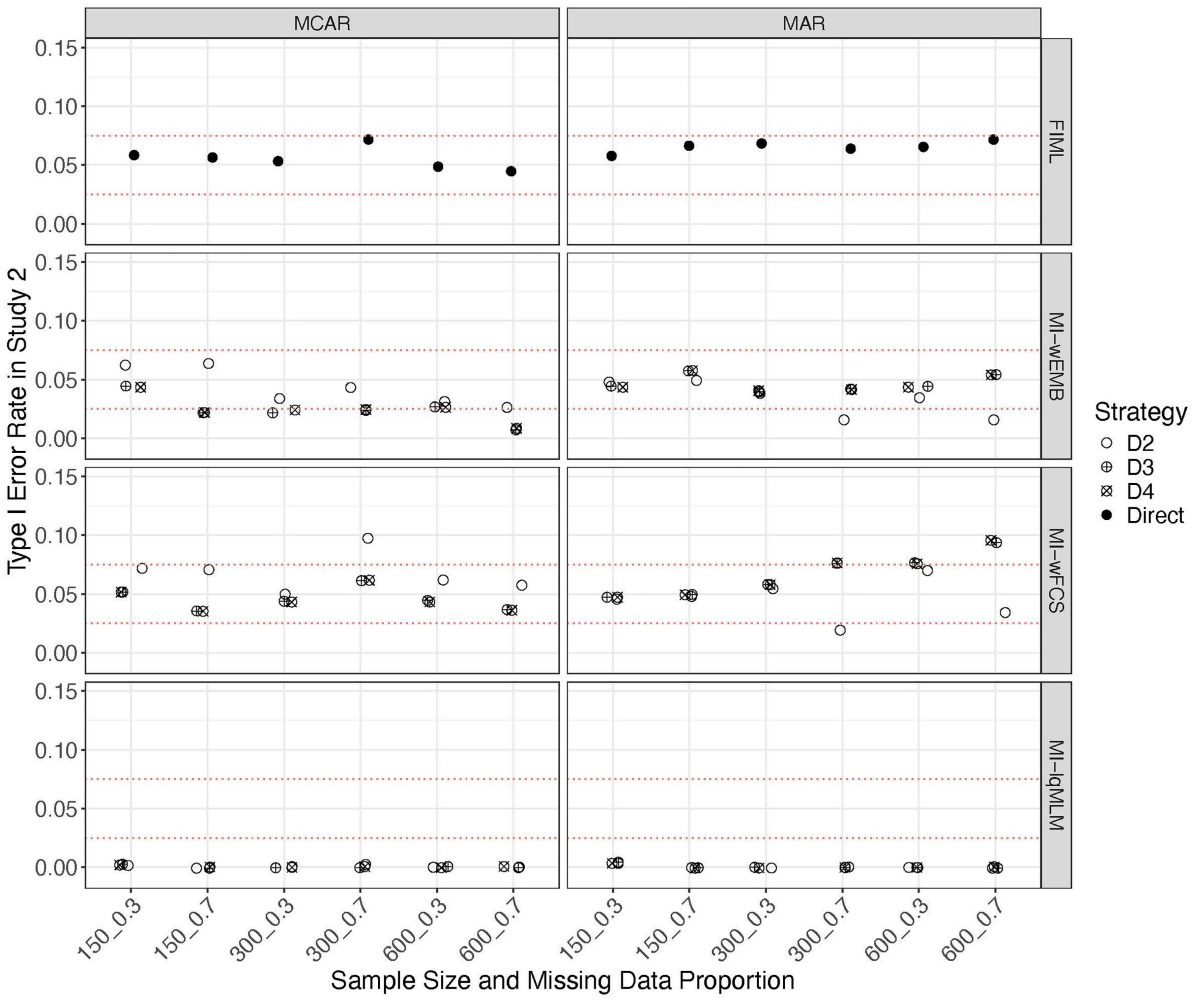
Study 1 results: type I error rates from missing data approaches for the linear model.

Figure 6 presents the Type I error rates for all approaches, including different pooling strategies. We found that the Type I error rates from FIML generally fell within the range of 0.025–0.075. Comparing the two wide-format imputation methods, MI-wEMB and MI-wFCS, the Type I error rates of test statistics pooled using D_2_, D_3_ and D_4_ were mostly within the acceptable range of 0.025–0.075 or hovered around its edges. MI-wEMB was more sensitive to conditions where the sample size was small (*N* = 150) and the proportion of missing data was large, whereas MI-wFCS demonstrated greater robustness in these challenging scenarios. The relative performance of the three pooling strategies was less clear. However, generally speaking, under MCAR conditions and with the two wide-format imputation methods, D_2_ tended to produce slightly higher Type I error rates compared to D_3_ and D_4_. Under MAR, D_2_ tended to generate slightly smaller Type I error rates than D_3_ and D_4_. Interesting but unsurprisingly, the two long-format imputation approaches, MI-llMLM and MI-lqMLM both yielded 0 Type I error rate, regardless of pooling approaches and design factors. This demonstrates a potential limitation of this imputation strategy in LGCM, which pre-assumes the shape of trajectory during imputation.

### 5.2 Study 2

#### 5.2.1 Parameter estimates

**Figure 7:**
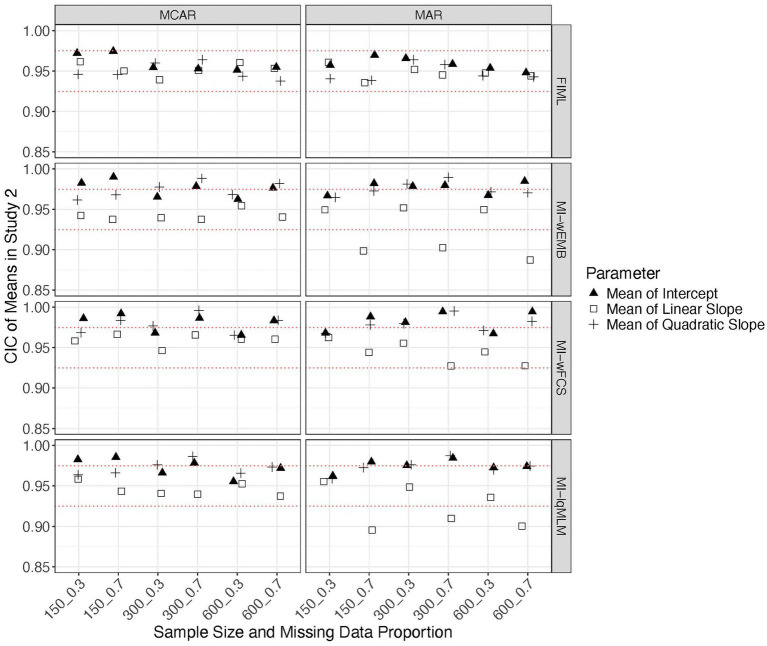
Study 2 results: CICs from missing data approaches for the latent means of in the quadratic model.

**Figure 8:**
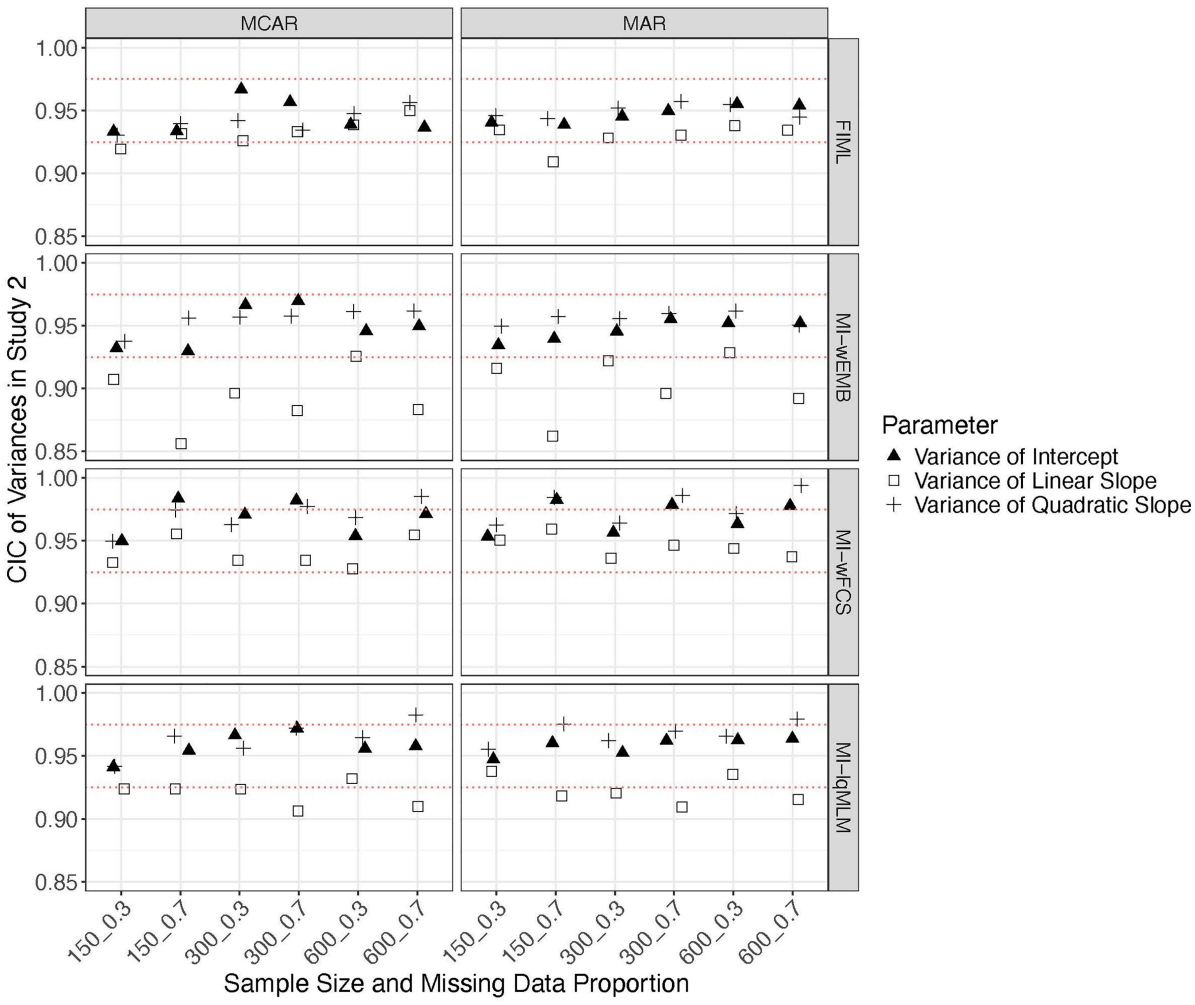
Study 2 results: CICs from missing data approaches for the latent variances of in the quadratic model.

**Figure 9:**
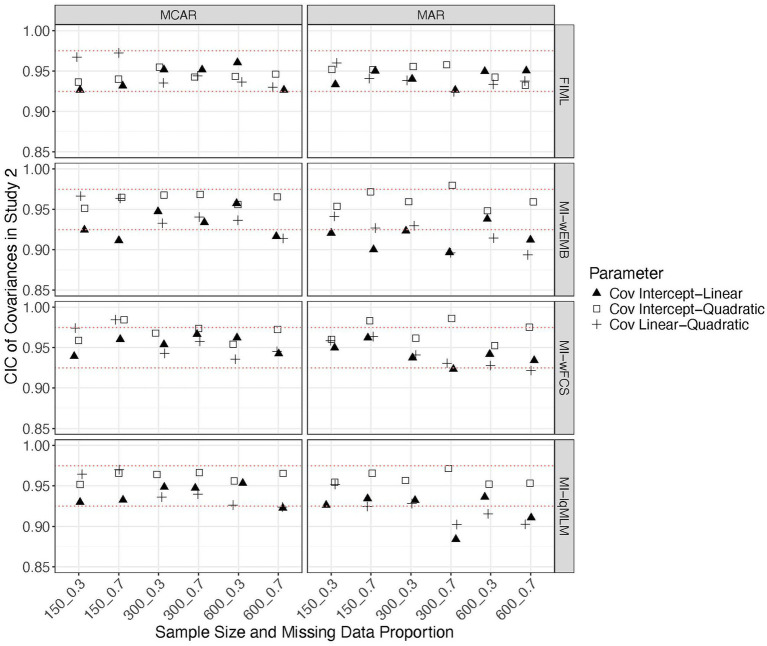
Study 2 results: CICs from missing data approaches for the latent covariances of in the quadratic model.

In this study we focus on a latent quadratic growth model. Similar to the findings from Study 1, the standardized biases from all methods fell within the acceptable range (i.e., > −0.4 and <0.4), except that for MI-wFCS the linear slope variance exceeded this range when the proportion of missing data was large. Overall, FIML and MI-lqMLM produced the most accurate parameter estimates. The MSEs from all methods were comparable across all conditions. A common pattern observed across methods was that when the sample size was small (*N* = 150), the MSEs for intercept variance were substantially larger than those for other parameters. The CICs were generally within the acceptable range (i.e., >0.90), with FIML yielding the most accurate CICs for nearly all parameters under most conditions. However, for MI-wEMB and MI-lqMLM, CICs associated with the latent linear slope (mean, variance, covariances) were heavily impacted by the proportion of missing data or missing data mechanism (see Figures 7–9). Specifically, for these methods, the CICs for the mean of the linear slope dropped below 0.925 or even below 0.90, under a larger proportion of MAR (Figure 7). The CICs for the variance of the linear slope were even lower for MI-wEMB (Figure 8). Additionally, for MI-wEMB and MI-lqMLM, overly small CICs were observed for covariances between the linear slope and both the intercept and quadratic slopes under a larger proportion of MAR (Figure 9). In contrast, MI-wFCS tended to generate higher CICs (>0.975) for parameters associated with the intercept and quadratic slopes.

#### 5.2.2 Type I error rate

**Figure 10:**
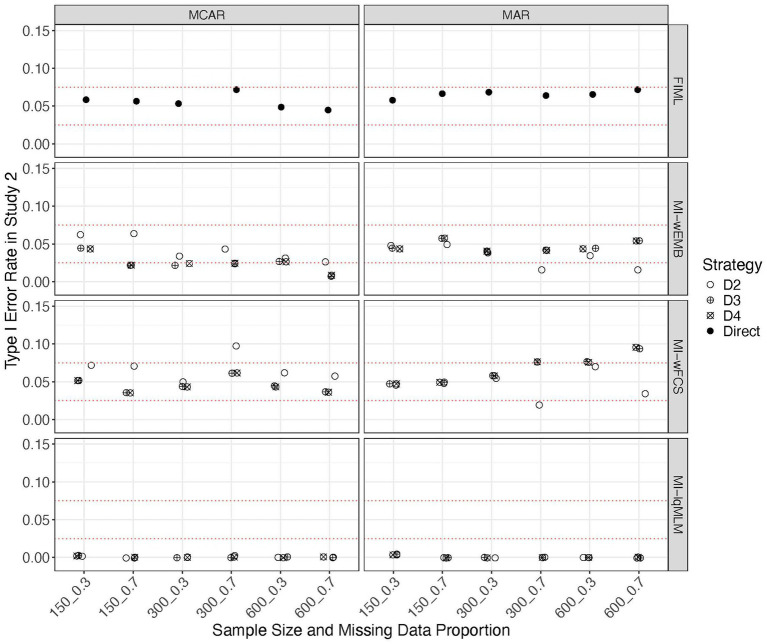
Study 2 results: type I error rates from missing data approaches for the quadratic model.

With the correctly specified analysis model, the Type I error rates for all methods remained <0.10. However, different patterns were observed (see Figure 10). FIML yielded the most accurate Type I error rates. Comparing between the two wide-format MI methods, MI-wEMB and MI-wFCS, the Type I error rates for test statistics pooled using D_2_, D_3_ and D_4_ fell within 0.025–0.075 or hovered around the edges, with only a few exceptions, particularly in cases with a large proportion of missing data. Similar to Study 1, the long-format MI method, MI-lqMLM, constantly produced Type I error rates of 0, regardless of pooling strategies or design factors.

### 5.3 Study 3

#### 5.3.1 Power

**Figure 11:**
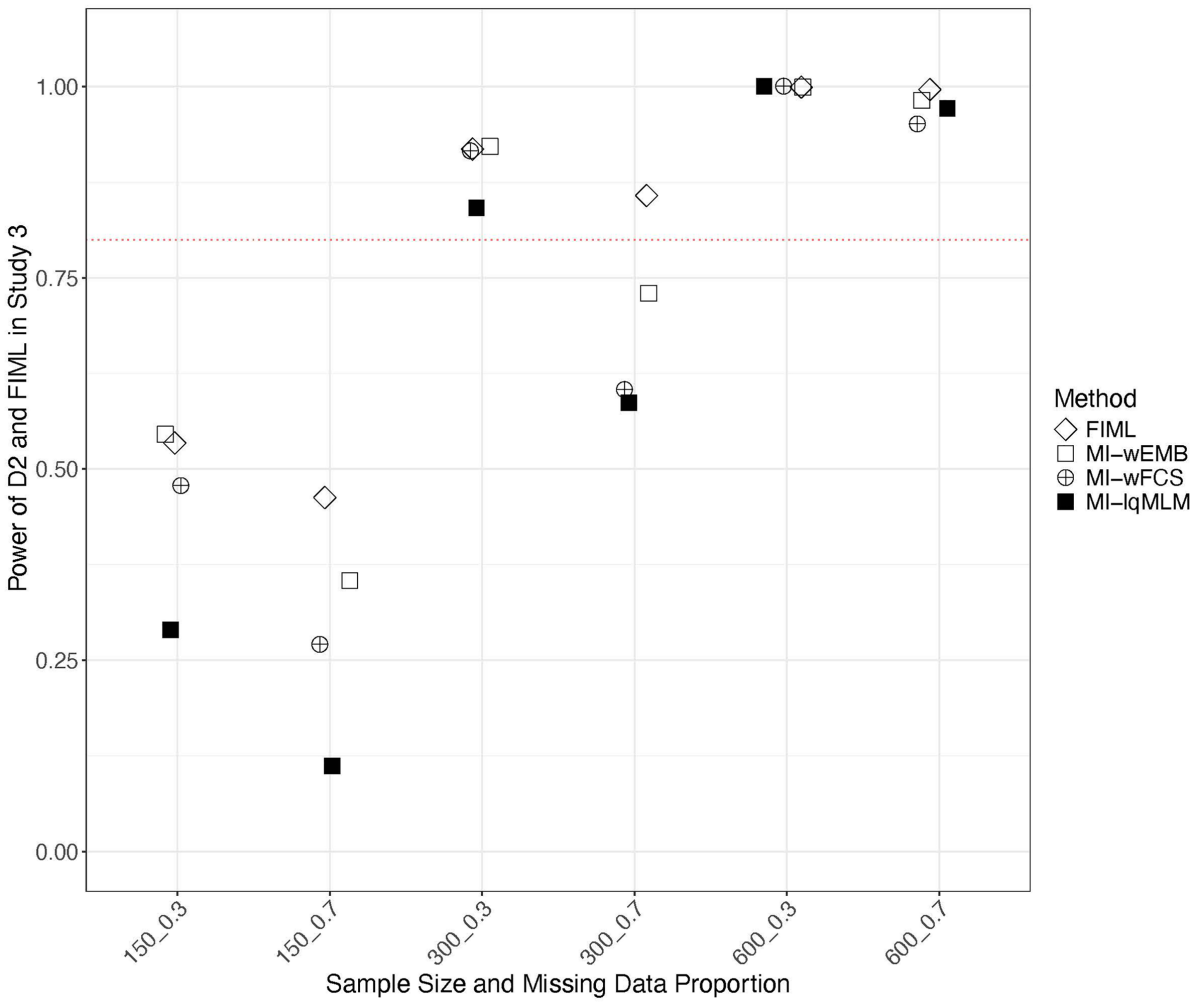
Study 3 results: power of D_2_ test statistic comparing with FIML in detecting misspecification in quadratic model.

**Figure 12:**
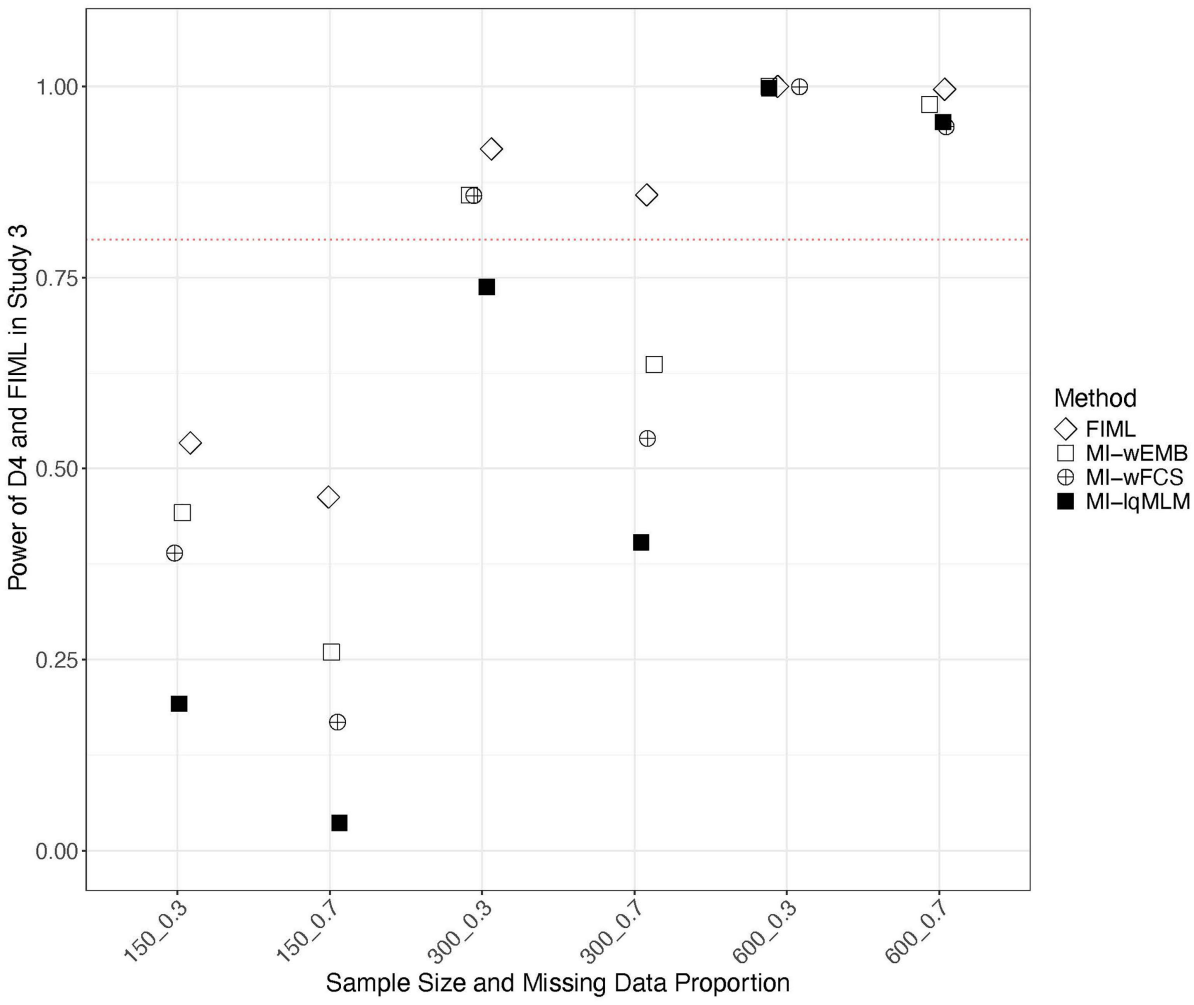
Study 3 results: power of D_4_ test statistic comparing with FIML in detecting misspecification in quadratic model.

In Study 3, we examined the power of various test statistics in detecting model misspecification under MAR. Figure 11 presents the power values from the D_2_ pooling strategy for three MI approaches (i.e., MI-wEMB, MI-wFCS and MI-lqMLM), compared to those from FIML. We found that FIML exhibited the highest power with the large proportion of missing data, followed by MI-wEMB, MI-wFCS and MI-lqMLM. The impact of sample size was also noticeable. Generally, for the same proportion of missing data, power increased as sample size grew. As sample size increased, power difference among FIML and MI approaches diminished significantly. Figure 12 presents a similar comparison using the D_4_ pooling strategy with FIML. While D_4_ tended to be more vulnerable to smaller sample sizes, it exhibited the same pattern observed with D_2_. Furthermore, with larger sample sizes, the power values for D_2_ and D_4_ converged. Power values from D_3_ were nearly identical to those with D_4_.

## 6 Empirical example

In this section, we applied the aforementioned imputation approaches and pooling strategies to a real-world data set for demonstration. The data were obtained from the Early Childhood Longitudinal Studies Program (ECLS-K: 2011 Kindergarten–Fifth Grade; Mulligan et al., 2019; Tourangeau et al., 2019). This national data set contains multisource information that offers a broad and multifaceted view of young children’s early childhood experiences at home and in school. The sample consists of children who either entered kindergarten for the first time or repeated kindergarten during the 2010–11 school year. We selected data collected during the fall semesters from kindergarten through second grade, and the spring semester from second grade through third grade. Among all the outcome measures related to children’s home and school experiences, our primary focus was on their trajectory of mathematics achievement. For demonstration purposes, we randomly selected 600 children who had complete data on their math item response theory (IRT) scores at all measurement points. We then imposed attrition under MAR at all time points except for the first one, following Equation 5, with b_1_ set at 0.4, and missing data proportions of five measurement occasions set at (0, 0.12, 0.24. 0.30, 0.36), respectively. At each time point, the probability of a value being missing is impacted by the values observed at the immediately preceding time point. We fit a quadratic latent growth curve model to the data. Then we intended to center time around the middle point and wrote loading matrix ( Λ ) as follows:


$$\Lambda =|\begin{array}{ccc} 1 & -1.5 & 2.25\\ 1 & -.5 & .25\\ 1 & 0.5 & .25\\ 1 & 1 & 1\\ 1 & 2 & 4 \end{array}|,$$

where the three columns represent the loadings for intercept, the liner slope and the quadratic slope, respectively. The analysis results are shown in the Table 2. The parameter estimates obtained from all MI methods were comparable to those from FIML.

**Table 2:** Results from empirical example: parameter estimate (standard error) and LRT statistic $\chi^{2}$

Parameter	Type	FIML	MI-wEMB	MI-wFCS	MI-lqMLM
Mean					
	Int.	0.339 (0.017)	0.382 (0.020)	0.340 (0.021)	0.346 (0.020)
	S1	0.784 (0.006)	0.783 (0.006)	0.784 (0.006)	0.784 (0.006)
	S2	−0.124 (0.003)	−0.124 (0.003)	−0.126 (0.003)	−0.126 (0.003)
Variance					
	Int.	0.262 (0.014)	0.261 (0.015)	0.264 (0.015)	0.264 (0.015)
	S1	0.015 (0.002)	0.015 (0.002)	0.015 (0.002)	0.015 (0.002)
	S2	0.003 (0.001)	0.003 (0.001)	0.003 (0.001)	0.003 (0.001)
Covariance					
	Int. with S1	−0.024 (0.003)	−0.028 (0.003)	−0.023 (0.003)	−0.024 (0.003)
	Int. with S2	0.000 (0.002)	0.000 (0.001)	0.000 (0.002)	0.001 (0.002)
	S1 with S2	−0.003 (0.001)	−0.004 (0.001)	−0.004 (0.001)	−0.004 (0.001)
$\chi^{2}$ (*df* = 6)		131.090			
	D_2_		131.304	120.319	75.281
	D_3_		125.178	125.217	70.161
	D_4_		125.177	125.219	70.161

Int., intercept; S1, linear slope of first phase; S2, linear slope of second phase.

In the empirical analysis, the LRT statistic evaluates the fit of the LGCM (null hypothesis) against the corresponding saturated model (alternative hypothesis). FIML yields the conventional model LRT statistic, whereas each multiple imputation method pools the LRT statistics generated from regular maximum likelihood across imputations using the D_2_, D_3_, and D_4_ pooling procedures. The D_2_ test statistics with MI-wEMB were most similar to that from FIML, while the D_3_ and D_4_ methods with MI-wEMB and all pooling methods with MI-wFCS yielded lower LRT statistics values. The lowest LRT statistic values were found from all pooling approaches with MI-lqMLM. These results were consistent with the simulation results from Study 2.

## 7 Discussion

The current study evaluated the efficacy of various MI approaches applied to wide- and long-format data in estimating parameters and assessing model fit in linear and quadratic latent growth curve models. Our aim was to offer researchers practical guidance for handling missing data within this specific modeling framework.

Our analysis revealed that, similar to FIML, the MI approaches consistently yielded unbiased parameter estimates and comparable MSEs cross all examined parameters. Specifically, all methods performed well in accurately estimating the means, variances, and covariances of the growth factors. However, divergent patterns emerged in CIC. For the linear growth model, CICs for the latent variance of the linear slope occasionally fell below 0.925 when using MI-wEMB and MI-lqMLM, particularly under higher levels of missingness in MAR conditions. For the quadratic model, larger proportions of missing data also led to overly liberal CICs (i.e., < 0.925) for MI-wEMB and MI-lqMLM, particularly for parameters related to the linear slope.

Model fit evaluation was one of the two central objects of our study. To assess model fit, we examined three pooled test statistics (D_2_, D_3_, and D_4_) obtained from the MI methods, and compared them with the FIML LRT statistic. When the analysis model was correctly specified, the Type I error rates of the pooled test statistics from the wide-format MI methods (MI-wEMB and MI-wFCS) were generally comparable to those produced by FIML. In contrast, the long-format MI methods (MI-llMLM and MI-lqMLM) yielded near-zero Type I error rates, indicating an overly conservative tendency. Under the quadratic model misspecification, where a latent covariance was incorrectly fixed to zero, MI-wEMB and MI-wFCS demonstrated the second- and third-highest power, respectively, to detect misspecification with smaller sample sizes, ranking just below FIML. As expected from the correct-model results, the long-format MI-lqMLM showed the lowest power. Further comparison under model misspecification revealed that the D_2_ test statistics from MI-wEMB produced power values comparable to FIML, whereas D_3_ and D_4_ consistently yielded lower power values.

Overall, unlike the wide-format imputation methods (MI-wEMB and MI-wFCS), the long-format methods (MI-llMLM and MI-lqMLM) performed poorly in model fit evaluation. This shortfall likely stems from the long-format methods’ transformation of data into long format prior to imputation, followed by the specification of a parametric linear (MI-llMLM) or quadratic (MI-lqMLM) mixed-effects model. These impose greater constraints than less parametric wide-format approaches (no transformation needed), which permits an unstructured covariance matrix across time points without assuming specific random-effects distributions (as in MI-wEMB), or imputes missing values in time points sequentially based on Bayesian regression (as in MI-wFCS). Consequently, the constrained imputation model in long-format methods compels imputed values to adhere closely to the assumed trajectory and clustering structure, thereby reducing unexplained variability in the completed datasets more than a less restrictive model would.

This study provides practical guidance for researchers to select the appropriate approach dealing with missing data in the context of the LGCM framework. Given the growing attention to multilevel long-format imputation methods in recent literature (e.g., Quartagno and Carpenter, 2019; Resche-Rigon and White, 2018; Audigier and Resche-Rigon, 2018; Enders et al., 2020; Grund et al., 2018), it is important to explore how these approaches can be effectively extended to the LGCM, particularly in addressing the inherent clustered data structure. Building on our findings, we highlight key insights as follows to support better methodological decision-making and identifying promising directions for future studies.

While our results indicate that FIML performs best under the specific conditions examined, this does not diminish the importance of MI in many real-world applications where FIML cannot be reliably implemented. MI is preferable in several key scenarios: when researchers require complete datasets for secondary analyses, encounter convergence issues with FIML (which are common in repeat measures designs), or need to incorporate auxiliary variables not included in the final analysis model. While MI’s stepwise approach yielded comparable results in most cases and offers greater flexibility, it requires careful specification of the imputation model and thoughtful selection of pooling strategies.

Despite differences observed in parameter estimates and model fit statistics, MI methods generally served as viable alternatives to FIML. All examined MI approaches generated unbiased parameter estimates, with MI-wFCS (implemented via Blimp) standing out for its robust performance, producing accurate CICs across all parameters and demonstrating minimal sensitivity to both the mechanism and proportion of missing data. MI-wEMB exhibited greater power in LRT overall than other MI methods. For model fit evaluation, the D_2_ test statistic paired with MI-wEMB most closely approximated FIML’s ability to detect misspecification in latent factor covariances, whereas D_3_ and D_4_ required larger sample sizes to perform adequately. Notably, as sample size increased, the difference in power among imputation and pooling methods diminished substantially.

The long-format multilevel imputation is particularly appealing for complex LGCMs with a large number of time points or nonlinear trajectories, as it can, in theory, account for within-person dependencies and between-person heterogeneity through the specification of random effects during the imputation phase. However, our findings under the examined conditions indicate that these theoretical advantages, primarily derived from the multilevel modeling framework, did not translate into clear practical benefits over standard wide-format imputation approaches in LGCM. The long-format imputation methods examined in this study showed outstanding performance in parameter recovery relative to their wide-format counterparts, however, they could be more sensitive to missing data proportion when estimating CIs. More critically, the long-format imputation methods associated with the D_2_, D_3_, or D_4_ pooling strategies produced near-zero Type I error rates. This result likely arose because the imputed values were generated deterministically and were overly consistent with the analysis model (e.g., linear or quadratic). Without valid between-imputation variability, these pooling strategies tended to underestimate the true uncertainty associated with the missing data. As a result, the pooled LRT statistics became overly conservative and were unlikely to reject the null hypothesis, even when they should have. This conservativeness not only deflated the Type I error rate but also severely reduced statistical power, limiting the ability to detect model misspecifications when they existed.

It is important to acknowledge the limitations of our findings within the context of the specific LGCMs and conditions examined. First, the conclusions may not generalize to alternative model configurations, such as conditional growth curve models with covariates, multivariate growth curve models, or multilevel latent growth curve models with additional hierarchical levels. Further research is needed to investigate these extensions. Second, the observed performance of MI methods may not hold across different types of model misspecifications. While our study focused on misspecification in the analysis model, misspecification can potentially arise in both the imputation and analysis models. The performance of long-format multilevel imputation under various misspecification scenarios presents an intriguing avenue for future investigation. Third, in our simulation, we focus exclusively on conditions allowing a straightforward head-to-head comparison of FIML and MI (e.g., no auxiliary variables under MCAR and few auxiliary variables under MAR), where both approaches are easily implemented and should be equally efficient in theory. However, as documented by Savalei and Bentler (2009), MI generally provides more straightforward inclusion of a large number of auxiliary variables, while FIML often requires saturated-correlates modeling with auxiliary variables that could substantially increase complexity and compromise convergence. This important practical distinction suggests avenues for future research, such as targeted comparisons of MI and saturated-correlates FIML in LGCMs with many auxiliaries or under conditions that challenge FIML convergence. Fourth, our simulation was conducted under the assumption of data normality, utilizing ML or FIML estimators. However, when normality is violated, methodological adjustments become critical. Nonnormality can introduce significant challenges, including biased standard errors, inaccurate test statistics, and potentially misleading fit indices. An important direction for future research involves exploring how different imputation and pooling strategies address nonnormality in LGCM. Fifth, our evaluation on long-format multilevel imputation was limited to the approaches available in Blimp (Enders et al., 2020; Keller and Enders, 2023) and did not include more recent or alternative substantive-model-compatible (SMC) imputation developments for multilevel data, such as the SMC-JM approach in the R package jomo (Quartagno and Carpenter, 2022; 2023), and the sequential modeling approach in the R package mdmb (Grund et al., 2021). Future research could incorporate direct comparisons of these methods under conditions similar to or more extreme than those examined in our study, to further inform methodological choices for handling complex missing data. Finally, the pooling strategies employed in this study did not appear to be well-suited for long-format multilevel imputation approaches. The observed deflated Type I error rates and reduced statistical power highlight the need for further methodological investigation and the development of more targeted post-imputation techniques.

## Data Availability

Publicly available datasets were analyzed in this study. This data can be found at: https://nces.ed.gov/ecls/dataproducts.asp. Further inquiries can be directed to the corresponding author.

## Footnotes

1 Complete results, including MSE, as well as the code for simulation and analysis in empirical example are available in OSF (https://osf.io/3bswx/overview).
